# The Small RNA RyhB Homologs from *Salmonella* Typhimurium Restrain the Intracellular Growth and Modulate the SPI-1 Gene Expression within RAW264.7 Macrophages

**DOI:** 10.3390/microorganisms9030635

**Published:** 2021-03-18

**Authors:** Diego Peñaloza, Lillian G. Acuña, M. José Barros, Paula Núñez, Fernanda Montt, Fernando Gil, Juan A. Fuentes, Iván L. Calderón

**Affiliations:** 1 Laboratorio de RNAs Bacterianos, Departamento de Ciencias Biológicas, Facultad de Ciencias de la Vida, Universidad Andres Bello, 8370186 Santiago, Chile; diego.ignacio.mp@gmail.com (D.P.); lillian.gabriela@gmail.com (L.G.A.); mbarrosgamonal@gmail.com (M.J.B.); paulanunezrojas@gmail.com (P.N.); fernanda.monttc@gmail.com (F.M.); 2 Microbiota-Host Interactions and Clostridia Research Group, Departamento de Ciencias Biológicas, Facultad de Ciencias de la Vida, Universidad Andres Bello, 8370186 Santiago, Chile; 3 ANID-Millennium Science Initiative Program-Millennium Nucleus in the Biology of the Intestinal Microbiota, 8370186 Santiago, Chile; 4 Laboratorio de Genética y Patogénesis Bacteriana, Departamento de Ciencias Biológicas, Facultad de Ciencias de la Vida, Universidad Andres Bello, 8370186 Santiago, Chile

**Keywords:** sRNA, RyhB paralogs, SPI-1, macrophage infection

## Abstract

Growing evidence indicates that small noncoding RNAs (sRNAs) play important regulatory roles during bacterial infection. In *Salmonella* Typhimurium, several sRNAs are strongly up-regulated within macrophages, but little is known about their role during the infection process. Among these sRNAs, the well-characterized paralogs RyhB-1 and RyhB-2 are two regulators of gene expression mainly related with the response to iron availability. To investigate the role of the sRNAs RyhB-1 and RyhB-2 from *S*. Typhimurium in the infection of RAW264.7 macrophages, we analyzed several phenotypic traits from intracellular mutant strains lacking one and both sRNAs. Deletion of RyhB-1 and/or RyhB-2 resulted in increased intracellular survival and faster replication within macrophages. The bacterial metabolic status inside macrophages was also analyzed, revealing that all the mutant strains exhibited higher intracellular levels of ATP and lower NAD^+^/NADH ratios than the wild type. Expression analyses from bacteria infecting macrophages showed that RyhB-1 and RyhB-2 affect the intra-macrophage expression of bacterial genes associated with the *Salmonella* pathogenicity island 1 (SPI-1) and the type III secretion system (T3SS). With a two-plasmid system and compensatory mutations, we confirmed that RyhB-1 and RyhB-2 directly interact with the mRNAs of the invasion chaperone SicA and the regulatory protein RtsB. Altogether, these results indicate that the RyhB homologs contribute to the *S*. Typhimurium virulence modulation inside macrophages by reducing the intracellular growth and down-regulating the SPI-1 gene expression.

## 1. Introduction

Once ingested, *Salmonella* Typhimurium must cope with several stress conditions during the passage from the stomach to the intestine. Subsequently, the pathogen can enter and proliferate within non-phagocytic and phagocytic cells, such as epithelial cells and macrophages, respectively. Inside macrophages, *S*. Typhimurium secretes effector proteins that generate a specialized intracellular compartment, the *Salmonella*-containing-vacuole (SCV). The SCV allows bacteria to evade macrophage killing, and these phagocytic cells become the vehicle for the systemic bacterial spread and the niche of persister cells [1,2]. In response to the stressful intracellular conditions, *S*. Typhimurium must quickly modulate its transcriptional profile, where the rapid gene expression regulation is relevant to a successful infection process. In this sense, bacterial sRNAs have emerged as important regulatory players under different physiological and stress conditions, including the infection process and the modulation of the microbial virulence in both Gram-negative and Gram-positive pathogens [3,4,5,6]. 

The intra-macrophage transcriptome of *S*. Typhimurium (strain 4/74) reported by Srikumar et al. (2015) [7] showed that the majority (88%) of the sRNAs identified in this bacterium are expressed within macrophages, and more than 30 of these are up-regulated compared to *S*. Typhimurium cultures at early stationary phase. This evidence suggests that these sRNAs could potentially play regulatory roles during infection and microbial virulence; however, little is known about this matter. The iron-responsive homologs RyhB-1 and RyhB-2 were the most highly up-regulated sRNAs within macrophages in this study, evidencing the iron-limited conditions inside these phagocytic cells [7]. RyhB-1 and its island-encoded paralog RyhB-2 from *S*. Typhimurium are two sRNAs well characterized by their role in iron homeostasis, and of which the expression is highly induced when bacteria are grown under conditions of iron starvation and during the infection of eukaryotic cells, such as fibroblast and macrophages [7,8,9,10]. In addition, their orthologs from *Salmonella* Typhi, RfrA and RfrB, are also known to be required for optimal intracellular replication within macrophages [11]. 

In previous studies, we demonstrated that the *S*. Typhimurium sRNAs RyhB-1 and RyhB-2 also participate in response to oxidative and nitrosative stress, suggesting that these sRNAs possess the ability to integrate a global response to multiple stresses encountered inside macrophages [12,13]. In a global RNA profiling from cultures of a *S*. Typhimurium strain overexpressing the island-encoded sRNA RyhB-2, we identified putative regulatory targets not only related with iron homeostasis and nitrosative stress but also with genes belonging to the *Salmonella* pathogenicity island 1 (SPI-1), the type III secretion system (T3SS), and some regulatory factors associated with the SPI-1 T3SS [13]. To investigate the regulatory roles of *S*. Typhimurium RyhBs during infection of murine macrophages, in the present study, we analyzed the intracellular survival, replication, and metabolic status of *S*. Typhimurium mutant strains lacking one or both sRNAs. Furthermore, the expression of some SPI-1-related genes was analyzed by quantitative RT-PCR in the mutant strains infecting macrophages. Our results provide evidence for the role of the sRNAs RyhB-1 and RyhB-2 from *S*. Typhimurium during the infection of murine macrophages. 

## 2. Materials and Methods

Bacterial strains and culture conditions. The strains and plasmids used in this study are listed in Table 1. The single (∆*ryhB-1* and ∆*ryhB-2*) and double mutant (∆*ryhB-1* ∆*ryhB-2*) strains of *S*. Typhimurium SL1344 (wild type, WT) were constructed by the technique of phage λ Red recombinase, as previously described [12,14]. The plasmids for arabinose inducible expression of RyhB-1 and RyhB-2 were constructed using the pBAD plasmid as previously described [15,16]. Briefly, for amplification of the *ryhB-1* and *ryhB-2* inserts, the respective forward primer starts with the sRNA +1 site and carries a 5′ phosphate modification. The respective reverse primer binds close to the 3′ end of the terminator sequence, including an XbaI site. The PCR products and vector were digested with XbaI and then ligated with T4 DNA ligase. The mutant versions of the plasmid-expressed RyhB-1 (pRyhB1^MUT1^ and pRyhB1^MUT2^) and RyhB-2 (pRyhB2^MUT1^ and pRyhB2^MUT2^) were generated by site-directed mutagenesis using overlapping PCR with appropriate primers as previously described [17]. For constitutive expression of *rtsB* and *sicA*, the pSF-p15A (Sigma**^®^**) vector was used, where the *rtsB* and *sicA* genes (including 5′ UTR regions) were cloned under the control of the *rpsL* promoter (30S ribosomal RNA) with appropriate primers including HindIII (forward) and EarI (reverse) restriction sites for *rpsL* cloning and EarI (forward) and XbaI (reverse) for *rtsB* and *sicA* cloning. The mutant versions of the plasmid-expressed RtsB (pRtsB^MUT^) and SicA (pSicA^MUT^) were generated by site-directed mutagenesis using overlapping PCR with appropriate primers as we previously described [17]. For the complementation assays, *ryhB-1* and *ryhB-2* were cloned with their respective native promoters into pBR322. The PCR fragments, generated using primers R1_pBR322_EcoRI_Fw/R2_pBR322_EcoRI_Fw and R1_pBR322_HindIII_Rev/R2_pBR322_HindIII_Rev, respectively, were digested with EcoRI and HindIII and subsequently cloned into pBR322 previously digested with the same enzymes to yield pPromRyhB1 and pPromRyhB2. All the primers used for these constructions are listed in Supplementary Table S1. 

Bacteria were grown routinely at 37 °C in Luria Bertani medium (LB) and aerated by shaking. When required, LB was supplemented with ampicillin (100 mg L^−1^), chloramphenicol (25 mg L^−1^), or kanamycin (50 mg L^−1^). Media were solidified by the addition of agar (15 g L^−1^). For growth curves, bacteria were grown in low magnesium minimal medium (LPM) at pH 5.8, and incubated at 37 °C with shaking. LPM contained 80 mM 2-(*N*-morpholino) ethanesulfonic acid (pH 5.8), 5 mM KCl, 7.5 mM (NH_4_)_2_SO_4_, 0.5 mM K_2_SO_4_, 0.1% casamino acids, 38 mM glycerol, 337.5 μM K_2_HPO_4_-KH_2_PO_4_ (pH 7.4), and 8 μM MgCl_2_.

Macrophage cells and intracellular proliferation assays. The RAW 264.7 murine macrophages (ATCC) were maintained in Dulbecco’s Minimal Essential Medium (DMEM) supplemented with 5% fetal bovine serum and incubated at 37 °C in 5% CO_2_. The infection assays were performed as previously described [7], with some modifications. RAW 264.7 macrophage cells were seeded in a 24-well plate (3 × 10^5^ per well) and infected with a DMEM bacterial suspension of complement-opsonized cells at a multiplicity of infection (MOI) of 100:1 (bacteria:macrophages). After 30 min of infection, the solution was removed, the cells were washed twice with PBS solution, and the supernatant was replaced with medium containing 100 μg mL^−1^ gentamycin. The antibiotic was left to act for 90 min, after which cells were washed twice with PBS solution before replacing the medium with medium containing 10 μg mL^−1^ gentamycin. At 2, 8, 12, and 24 h post-infection (hpi), the medium was removed, cells were washed twice with PBS, and resuspended in a PBS solution of 0.05% sodium deoxycholate. The bacterial suspension was serially diluted, plated on LB agar, and incubated overnight at 37 °C to count cell forming unit (CFU).

Measurement of intracellular bacterial replication by fluorescence dilution. The procedure was performed as previously described [18], with some modifications. Bacterial strains carrying pDiGc plasmid [19] were grown overnight in LPM at 37 °C with aeration and supplemented with 4% arabinose to allow production and maturation of red, fluorescent protein. Bacteria (1 × 10^7^ cells) producing red, fluorescent proteins were purified by cell sorting using FACSAria III (Becton Dickinson) to infect RAW 264.7 macrophage cells as described above. PBS suspensions of bacteria released from RAW 264.7 macrophage cells were analyzed using a FACSCalibur cytometer (Becton Dickinson) for fluorescence intensities in FL-1 and FL-2 channels. Data were analyzed with FlowJo 8.6.3 software. The pDiGc was a gift from Sophie Helaine and David Holden (Addgene plasmid # 59322; http://n2t.net/addgene:59322 (accessed on 27 February 2021); RRID:Addgene_59322).

Fluorescence microscopy. RAW 264.7 macrophage cells previously infected with bacteria expressing GFP (carrying pDiGc plasmid) were fixed in 4% paraformaldehyde in PBS pH 7.4 for 30 min with agitation at room temperature and then washed three times in PBS. To stain the nuclei of RAW 264.7 macrophage cells, Hoescht 33342 was added for 15 min and washed twice in PBS. Coverslips were mounted with a drop (10 µL) of Fluoromount-G™ (Invitrogen) on glass slides, and then the cells were visualized in a Leica TCS SP8 microscope. 

**Table 1:** Bacterial strains and plasmids used in this study.

	Relevant Characteristic (s)	Reference/Source
**Strain**		
WT	Wild type strain of *S*. Typhimurium SL1344	[20]
∆*ryhB-1*	*S*. Typhimurium SL1344 lacking *ryhB-1* gene	This study
∆*ryhB-2*	*S*. Typhimurium SL1344 lacking *ryhB-2* gene	This study
∆*ryhB-1* ∆*ryhB-2*	*S*. Typhimurium SL1344 lacking *ryhB-1* and *ryhB-2* genes	This study
*Escherichia coli* JM109	Strain used for heterologous expression analyzes by two-plasmid systems	Promega^®^
**Plasmid**		
pBR322	ApR, TcR, ColEl Ori	New England Biolabs^®^
pPromRyhB1	*ryhB-1* region of *S*. Typhimurium cloned into pBR322	This study
pPromRyhB2	*ryhB-2* region of *S*. Typhimurium cloned into pBR322	This study
pDiGc	bla GFP pBAD DsRed ori f1 AmpR	[19]
pBAD-His-Myc A	pBAD expression plasmid, ApR, pBR322 Ori	Invitrogen^®^
pRyhB1	pBAD-RyhB1 vector, arabinose inducible	This study
pRyhB1^MUT1^	pBAD-RyhB1 with *ryhB-1* mutated at positions 43–48	This study
pRyhB1^MUT2^	pBAD-RyhB1 with *ryhB-1* mutated at positions 52–55	This study
pRyhB2	pBAD-RyhB2 vector, arabinose inducible	This study
pRyhB2^MUT1^	pBAD-RyhB2 with *ryhB-2* mutated at positions 44–49	This study
pRyhB2^MUT2^	pBAD-RyhB2 with *ryhB-2* mutated at positions 53–56	This study
pSF-p15A	pSF-CMV-p15A Ori vector, KmR, p15A Ori	Sigma^®^
pSFp15A-*rpsL*	pSF-p15A vector with the *rpsL* promoter	This study
pRtsB	pSFp15A-*rpsL* vector with *rtsB* (from −20, relative to AUG) under the control of *rpsL* promoter (30S ribosomal RNA) for its constitutive expression	This study
pRtsB^MUT^	pRtsB vector with *rtsB* mutated at positions -1 and -5, relative to AUG	This study
pSicA	pSFp15A-*rpsL* vector with *sicA* (from its transcriptional start site) under the control of *rpsL* promoter (30S ribosomal RNA) for its constitutive expression	This study
pSicA^MUT^	pSicA vector with *sicA* mutated at positions -1 and -4, relative to AUG	This study

Intracellular ATP levels and NAD^+^/NADH ratios. ATP levels and nicotinamide nucleotide quantification were measured with the ATP Fluorometric Assay Kit and NAD^+^/NADH quantification kit, respectively (BioVision Research Products, Milpitas, CA, USA), according to the manufacturer’s instructions. Bacteria were released from RAW 264.7 macrophage cells at 8 hpi, washed twice with cold, sterile phosphate-buffered saline (PBS; pH 7.2), and then lysed with the extraction buffers. The samples were centrifuged (16,000× *g* for 3 min) to collect the supernatant. The measurements of ATP and NAD^+^/NADH were normalized to CFU values. 

RNA extraction and Real-Time PCR. Total RNA extraction was performed by the acid phenol method as previously described [17] from bacteria released from RAW 264.7 macrophage cells at 8 hpi, or from bacteria grown in LB until exponential phase (OD_600_ of 0.3). The qRT-PCR analyzes were performed as previously described [12]. Briefly, Real-time PCR was performed using the AriaMx Real-Time PCR System (Agilent Technologies Japan). The reaction mixture (10 μL) comprised 2 μL cDNA, 1 × KAPA SYBR^®^ FAST qPCR Master Mix (KAPABiosystems.), 0.25 μmol of each primer, and water. The PCR conditions were 95 °C for 3 min followed by 95 °C for 3 s, 60 °C for 15 s, and 72 °C for 10 s for 40 cycles. Melting curves (1 °C steps between 60 and 95 °C) ensured that a single product was amplified in each reaction. Real-time PCR data were analyzed using the 2^−ΔΔCT^ method to calculate the relative levels of gene expression in mutant strains and were expressed as relative expression (fold change) with respect to the wild type strain. The 16S gene was used for housekeeping gene. Results were expressed as an average of three independent replicates with the corresponding standard deviation. Specific primers used are listed in Supplementary Table S1.

Statistics. *p* values were calculated according to the Student’s *t*-test. Values of *p* < 0.05 were considered statistically significant.

## 3. Results

### 3.1. S. Typhimurium sRNAs RyhB-1 and RyhB-2 Regulate Intracellular Proliferation in Murine Macrophages

**Figure 1:**
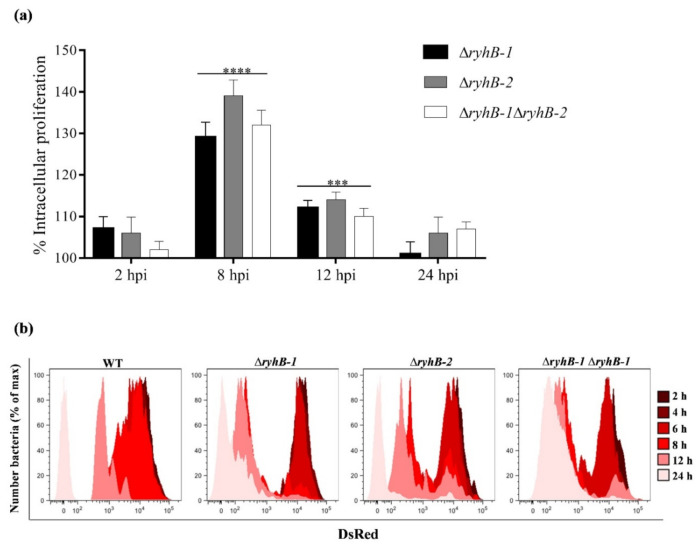
Intracellular proliferation of *S.* Typhimurium *ryhB*s mutants. RAW264.7 macrophages were infected with wild type (WT), ∆*ryhB-1*, ∆*ryhB-2*, and ∆*ryhB-1* ∆*ryhB-2* strains. (**a**) The number of intracellular bacteria was determined at the indicated hour post-infection (hpi) by plating serial dilutions of RAW264.7 lysates on Luria Bertani (LB) plates for counting CFU. CFU were counted in the initial inoculum and at the different hpi. CFUs were expressed as a percentage of the intracellular proliferation respect to the wild type (100%). Data represent the means ± standard deviations (*n* = 3) (*** *p* = 0.0001; **** *p* < 0.0001). (**b**) *S.* Typhimurium replication in RAW264.7 macrophages determined by flow cytometric detection of DsRed and EGFP fluorescence at different hpi from the bacterial strains carrying the pDiGc plasmid (*n* = 30,000 events analyzed at each time point).

Since the expression of both RyhB-1 and RyhB-2 is highly induced inside murine macrophages [7,8], thus suggesting that they could potentially play a regulatory role during infection, we first examined the effect of the sRNAs on the *S*. Typhimurium proliferation into murine RAW264.7 macrophages. For this purpose, the wild type, single (∆*ryhB-1* and ∆*ryhB-2*), and double mutant (∆*ryhB-1* ∆*ryhB-2*) strains of *S*. Typhimurium SL1344 were used to infect the RAW264.7 macrophage cell line. The intracellular bacterial survival was determined by counting CFU at 2, 8, 12, and 24 h post-infection in a gentamicin assay. All the mutant strains showed a significantly higher survival than the wild type strain inside macrophages at 8 and 12 h post-infection, with the more pronounced phenotype at 8 h post-infection (Figure 1a), coinciding with the time in which these sRNAs are more induced inside macrophages as previously reported by Padalon-Brauch et al. (2008) [8]. Under our experimental conditions, we confirmed that the expression of both RyhB-1 and RyhB-2 is highly induced inside RAW264.7 macrophages at 8 h post-infection (Figure S1). The fact that the wild type strain exhibits the lowest proliferation phenotype within macrophages, and that the expression of neither sRNA alone was sufficient to inhibit the intracellular proliferation in the single mutant strains indicates that both sRNAs contribute to a proliferation arrest of intracellular bacteria, with not redundant roles. 

The bacterial replication within macrophages was accurately determined by a fluorescence dilution (FD) assay based on a replication reporter system. This approach uses two fluorescent reporter proteins, a green constitutive for tracking bacteria and a red inducible to monitor the bacterial replication due to dilution of the red fluorescence because of bacterial cell division [19]. The FD results were in accordance with the previously CFU determination, showing that the Δ*ryhB-1*, Δ*ryhB-2,* and Δ*ryhB-1* Δ*ryhB-2* strains replicate at a much faster rate than the wild type, displaying a noticeable dilution of red fluorescence already at 8 h post-infection (Figure 1b). Fluorescence microscopies with *gfp*-expressing bacteria inside RAW264.7 macrophages at 8 hpi also allowed us to visualize the ability of the mutant strains to replicate faster than the wild type (Figure S2). 

### 3.2. RyhB-1 and RyhB-2 Deletions Result in Metabolically More Active Intracellular Bacteria

**Figure 2:**
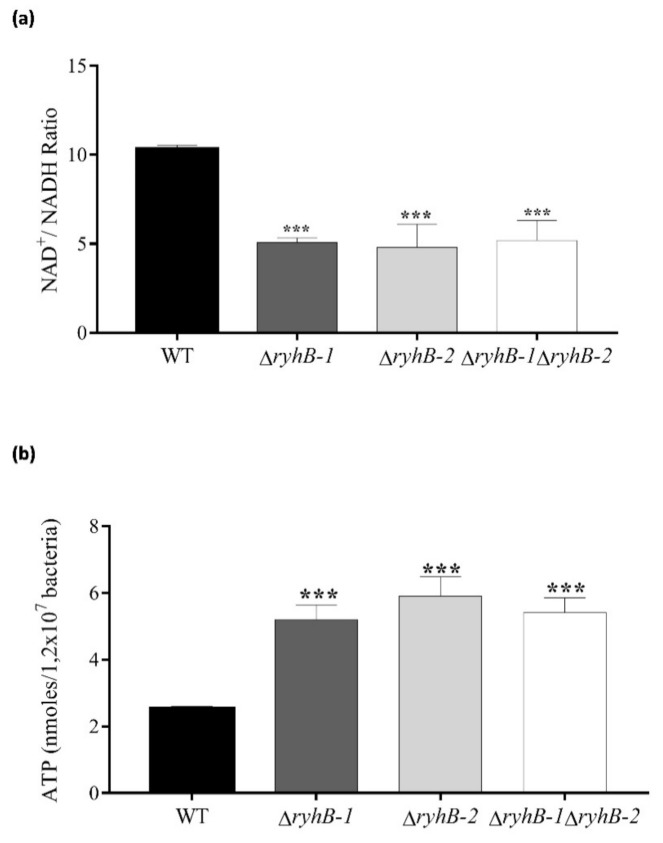
Metabolic status of intracellular *S.* Typhimurium *ryhB*s mutants. RAW264.7 macrophages were infected with wild type (WT), ∆*ryhB-1*, ∆*ryhB-2*, and ∆*ryhB-1* ∆*ryhB-2* strains, and both the NAD^+^/NADH ratio (**a**) and ATP levels (**b**) were determined from intracellular bacteria at 8 hpi. Asterisks represent statistical differences with respect to the wild type strain (*** *p* = 0.0001). Data represent the means ± standard deviations (*n* = 3).

Since the strains lacking one or both sRNAs exhibited higher proliferative phenotypes inside macrophages, and it is known that these sRNAs down-regulate the expression of iron-containing enzymes related with energy metabolism, we analyzed the metabolic status of the intracellular *ryhB*s mutant strains at 8 h post-infection, the time of higher RyhBs expression [8], as well as the time of more differentiable proliferative phenotypes here observed. We firstly analyzed the intracellular NAD^+^/NADH ratios from the strains infecting macrophages at 8 h post-infection (Figure 2a). We found that the Δ*ryhB-1*, Δ*ryhB-2*, and ∆*ryhB-1* ∆*ryhB-2* strains show the lowest NAD^+^/NADH ratios, similar among them, but differing from the wild type strain, which exhibited a higher NAD^+^/NADH ratio. To further confirm that the absence of the sRNAs resulted in a more active metabolic state, we measured the major energy currency of cells, i.e., the ATP levels, from bacteria inside macrophages. As expected, the bacterial levels of ATP in the strains lacking one or both RyhBs were higher than that of the wild type (Figure 2b). Together, these results indicate a more reductive intracellular environment in the mutant strains infecting macrophages and the subsequent higher intracellular levels of ATP, revealing that the absence of the sRNAs induces a more active metabolic state.

### 3.3. RyhB-1 and RyhB-2 from S. Typhimurium Affect the Expression of Genes Related to SPI-1 and Metabolism during Infection of RAW264.7 Macrophages 

**Figure 3:**
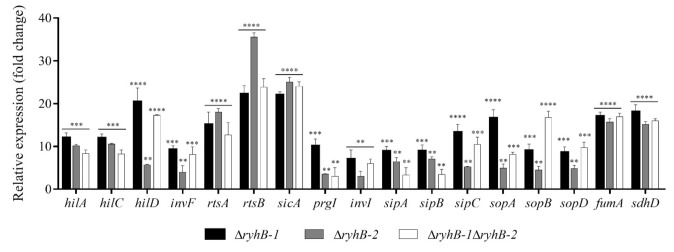
Expression of SPI-1- and metabolism-related genes from intracellular bacteria infecting RAW264.7 macrophages. RAW264.7 macrophages were infected with wild type, ∆*ryhB-1*, ∆*ryhB-2*, and ∆*ryhB-1* ∆*ryhB-2* strains. Total RNA was extracted at 8 hpi and the relative expression of putative targets was determined from intracellular bacteria by qRT-PCR. Values were normalized to the levels of the 16S rRNA. The relative expression of each mRNA transcript of the mutant strains was calculated using the 2^−ΔΔCT^ method and represented as the *n*-fold change relative to the wild type strain. Asterisks represent statistically significant differences with respect to the wild type (** *p* < 0.001; *** *p* = 0.0001; **** *p* < 0.0001). Data represent the means ± standard deviations (*n* = 3).

In a previous study on characterizing the role of RyhB-1 and RyhB-2 in response to nitrosative stress, we performed a global RNA profiling from cultures of a *S*. Typhimurium strain overexpressing the island encoded sRNA RyhB-2 [13]. Among the significantly down-regulated genes upon the pulse-expression of RyhB-2, we found several related to the SPI-1 T3SS and its regulatory network. Based on this information, here, we analyzed the expression of SPI-1-associated genes and known targets of RyhBs involved in energy metabolism from bacteria infecting macrophages. The transcript levels were determined by Real-Time PCR (qRT-PCR) from wild type, Δ*ryhB-1*, Δ*ryhB-2,* and Δ*ryhB-1* Δ*ryhB-2* strains infecting macrophages at 8 h post-infection. Interestingly, all the SPI-1-related genes analyzed exhibited significant up-regulation, to different extents, in both single and double deletion backgrounds, indicating that both RyhB-1 and RyhB-2 negatively affect their expression (Figure 3). The genes whose expression was affected were those encoding the transcriptional regulators HilA, HilC, HilD, InvF, and RtsA, the regulatory protein RtsB, the invasion chaperone SicA, the needle proteins PrgI and InvI, the effector proteins SipA, SipB, and SipC, and the SPI-1-associated genes encoding for SopA, SopB, and SopD effector proteins. The tricarboxylic acid (TCA) cycle genes *fumA* and *sdhD* were also up-regulated in the Δ*ryhB* backgrounds (Figure 3). Complementation of Δ*ryhB-1* and Δ*ryhB-2* restored the expression levels to those observed in the wild type strain, confirming that the gene expression changes were due to the deletion of the *ryhB* genes rather than a polar effect of the mutation (Figure S4). Taken together, these results indicate that both sRNAs contribute to the repression of SPI-1-related genes, known to be mainly involved in *Salmonella* host cell invasion during the early phase of infection [21], and the down-regulation of genes encoding iron-containing enzymes involved in energy metabolism in the intra-macrophage environment.

### 3.4. The RyhB sRNAs Directly Interact with the sicA and rtsB mRNAs 

**Figure 4:**
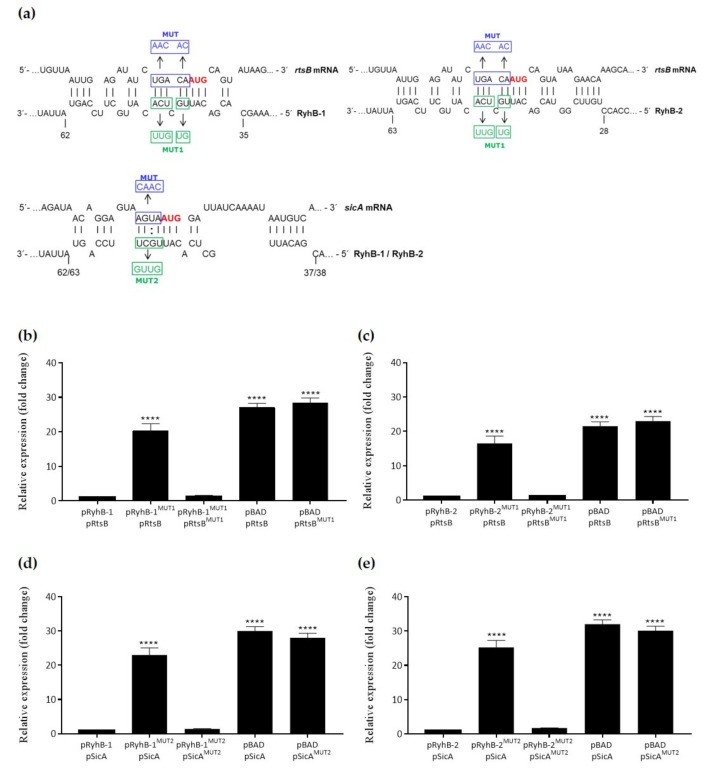
RyhB-1 and RyhB-2 down-regulate the *rtsB* and *sicA* expression by base-paring. (**a**) Schematic representation of the proposed interactions between the sRNAs RyhBs and *rtsB* and *sicA* mRNAs, including the nucleotide substitutions and compensatory mutations in blue boxes for the mRNAs and green boxes for the sRNAs, and the translational start sites highlighted in red. The numbers under the sRNA sequences indicate the base positions in the RyhBs. (**b**–**e**) Heterologous expression analyzes by two-plasmid systems. *E. coli* cells harboring the constitutive-expression plasmids pRtsB or pSicA plus the arabinose-inducible plasmids pRyhB-1 or pRyhB-2 were grown to exponential phase, and the expression of sRNAs was induced for 30 min by adding 0.2% arabinose. Total RNA was extracted and the relative expression of *rtsB* and *sicA* was analyzed by qRT-PCR. When required, *E. coli* cells were cotransformed with the corresponding mutant versions: pRtsB^MUT^ or pSicA^MUT^ plus the pRyhB1^MUT1^, pRyhB2^MUT1^, pRyhB1^MUT2^, or pRyhB2^MUT2^ plasmids. pBAD corresponds to the control empty plasmid. Values were normalized to the levels of the 16S rRNA. The relative expression of each mRNA transcript in the mutant versions was calculated using the 2^−ΔΔCT^ method and represented as the *n*-fold change relative to the wild type versions. Asterisks represent statistically significant differences (**** *p* < 0.0001). Data represent the means ± standard deviations (*n* = 3).

We look for direct targets of RyhB-1 and/or RyhB-1 by in silico analyses with the IntaRNA program [22,23,24], to predict potential interactions between the sRNAs and the Shine-Dalgarno region of the mRNAs here analyzed. Accordingly, both RyhB-1 and RyhB-2 were predicted to interact with relatively long sequences of the *rtsB* and *sicA* mRNAs around the 5′ untranslated region (5′ UTR) and the start codons (Figure 4a). To investigate this further, we used a two-plasmid system in a heterologous background (*E. coli* JM109), wherein *rtsB* or *sicA* were constitutively produced from a plasmid (pRtsB and pSicA), and RyhB-1 and RyhB-2 were overexpressed for 30 min from arabinose-inducible plasmids (pRyhB-1 and pRyhB-2, respectively). We also introduced mutations and compensatory mutations in all the plasmid constructions in the complementary regions of the targets and the sRNAs to disrupt and restore the sRNA-mRNA interactions, respectively (Figure 4a). Base changes introduced into the complementarity regions of the plasmid-expressed sRNAs affected the expression of *rtsB* (Figure 4b,c) and *sicA* (Figure 4d,e). The base changes into RyhB-1 and RyhB-2, between bases 43–48 (pRyhB1^MUT1^) and 44–49 (pRyhB2^MUT1^), respectively, strongly reduced the negative effect on the *rtsB* expression, while the compensatory mutations into *rtsB* between positions -1 and -5 (pRtsB^MUT^), with respect to AUG start codon, resulted in expression levels similar to that of fully wild-type genes (Figure 4a–c). For its part, the base changes into RyhB-1 and RyhB-2 at positions 52–55 (pRyhB1^MUT2^) and 53–56 (pRyhB2^MUT2^), respectively, reduced the negative effect on *sicA* expression. By contrast, the compensatory mutations into *sicA* at positions -1 and -4 (pSicA^MUT^), concerning the AUG start codon, restored the repressive effect on *sicA* expression to that of fully wild-type genes (Figure 4d,e). When arabinose was omitted, the observed repressive effects on the expression of target genes were abolished (data not shown). These results indicate that RyhB-1 and RyhB-2 down-regulate the *rtsB* and *sicA* mRNA levels by direct interaction.

## 4. Discussion

In the last two decades, bacterial sRNAs have emerged as important post-transcriptional regulatory molecules required for the adaptation to different stress conditions to fine-tuning physiological processes, such as quorum sensing, biofilm formation, intermediate metabolism, and virulence [6,25,26,27]. Several studies have demonstrated the expression of specific sRNAs by intracellular *S*. Typhimurium located inside eukaryotic cells [7,9,28,29]; however, the functions of these sRNAs along the intracellular infection cycle remain poorly characterized. In the present study, we investigated the contribution of the sRNAs RyhB-1 and RyhB-2 from *S*. Typhimurium during the infection of RAW264.7 macrophages, a cell type with the ability to destroy microorganisms but paradoxically the vehicle for systemic bacterial spread [2,30]. By analyzing the intracellular survival, replication, and metabolic status of *S*. Typhimurium mutant strains lacking one or both sRNAs, we demonstrated that RyhB-1 and RyhB-2 contribute to restrain intracellular bacterial growth. Furthermore, we noticed that the intracellular expression of SPI- and energy metabolism-related genes are affected by both sRNAs. 

As aforementioned, the RyhB-1 and RyhB-2 levels increase in intracellular *S*. Typhimurium in both fibroblasts and macrophages [7,8,9], suggesting that they could play a regulatory role during the infection of these eukaryotic cells. In our analysis, we observed that both RyhB-1 and RyhB-2, individually or together, contribute to attenuate intracellular bacterial growth, conversely to that previously reported in fibroblasts where only the absence of both together, i.e., the double mutant, was required to restrain the intracellular bacterial growth [9]. These dissimilar observations probably reflect the differential regulatory roles of these sRNAs related to the different intracellular conditions existing between the phagocytic and non-phagocytic cells. In the case of macrophages, bacteria must overcome an extensive antimicrobial arsenal, including oxidative and nitrosative stress, being able to replicate and even persist in this harsh environment [30]. In response to both conditions, oxidative and nitrosative stress, the participation of RyhB-1 and RyhB-2 has been previously demonstrated from extracellular cultures of *S*. Typhimurium [12,13]. In this sense, the bacterial growth restraint mediated by RyhB-1 and RyhB-2 is probably a strategy of *S*. Typhimurium to avoid an exacerbated activation of innate and/or adaptative immune responses, thus allowing the pathogen to spread and persist. It is known that the intracellular proliferation is essential for the virulence of *S*. Typhimurium [31]. Nevertheless, different studies have revealed that *Salmonella* presents several responses directed to down-regulate the intracellular proliferation, indicating that this may be a mechanism of immune evasion in order to enhance virulence [32]. Albaghdadi et al. (2009) [33] demonstrated that a reduced intracellular proliferation limits antigen presentation and development of a rapid CD8^+^ T cell response, reinforcing this hypothesis. For its part, Eriksson et al. (2000) [34] identified that *S*. Typhimurium mutants with overgrowth phenotypes within the macrophage are attenuated for virulence.

In accordance with the growth phenotypes observed in intracellular conditions, we also observed that deletions of RyhB-1 and RyhB-2 induce an increased bacterial growth in extracellular cultures in low magnesium minimal medium (Figure S3), similar to what was previously observed by Padalon-Brauch et al. (2008) [8] with *ryhB* mutant strains cultured under iron-deprived conditions. These two conditions are encountered within macrophages and produce *S.* Typhimurium transcriptomic profiles that resemble the intra-macrophage stage [7,35,36]. The fact that the wild type strain exhibits the lowest proliferation phenotype within macrophages and extracellular cultures, and that the expression of neither sRNA alone was sufficient to inhibit the proliferation in the single mutant strains indicates that both sRNAs contribute to a growth arrest of bacteria with non-redundant roles. 

Regarding the more active intracellular metabolic status observed in the *ryhB*-knockout mutants, this finding argues in favor of their intracellular proliferative phenotypes and can be explained by the targets that are known to be down-regulated by these sRNAs, in *S*. Typhimurium and other bacteria, related with genes encoding enzymes from the TCA cycle, among others [10,13,18,37,38]. The absence of the RyhB-1 and RyhB-2 provokes the loss of the negative control on these targets, and probably, this more active metabolic state observed (Figure 2). It should be noted that enzymes known to be expressed in aerobically growing cells, such as encoded by the succinate dehydrogenase operon, are also induced within macrophages, indicating that oxygen is freely available in the SCV [39]. Furthermore, the overall in vivo expression profile of *S*. Typhimurium suggests that aerobic respiration was occurring inside macrophages [39]. Considering that these metabolic targets include iron-containing enzymes, its expression in a controlled manner allows bacteria to carry out aerobic respiration, optimize the use of this scarce element inside the host, and enable the pathogen to limit the oxidative and cytotoxic effects associated with the Fe^2+^ released from these and other proteins upon the damage produced by the oxidative and nitrosative burst of macrophages [40,41,42]. Thus, the gene expression modulation of iron-containing proteins results essential for successful infection of *S*. Typhimurium and many pathogens, and the role of the RyhB sRNAs could be crucial for this purpose.

Most genetic approaches directed to define the function of sRNAs rely on pulsed ectopic expression of the sRNA in bacteria grown in culture media to identify sRNA targets experimentally; however, this procedure might not be suitable when the aim is to characterize the regulatory role of an sRNA during the infection [43]. In the infection process, bacteria are subjected to diverse stresses, where the macrophages contribute with several antimicrobial mechanisms [36,44]. Accordingly, the transcriptome undergoes a dynamic remodeling for bacterial adaptation when compared to bacteria grown in axenic cultures [29,43,45,46]. Therefore, the pulsed expression of an sRNA up-regulated intracellularly from bacteria growing extracellularly might be misleading in identifying the cellular targets. To overcome this issue, and based on our previous study in which the pulsed ectopic expression of RyhB-2 in axenic cultures identified virulence genes significantly affected, we performed qRT-PCR analyses in the wild-type and sRNA-defective bacteria isolated from RAW264.7 macrophages. We observed that the expression of the TCA cycle enzymes succinate dehydrogenase and fumarase A was increased in the absence of one or both sRNAs, which could explain, at least in part, the more active metabolic state in the *ryhB*-knockout mutants because of loss of negative control on these TCA enzymes. The expression of *fumA* and *sdhD* is directly regulated by RyhB in *E. coli* [37]; hence, we infer that this mechanism is conserved *S*. Typhimurium. 

The function of SPI-1 is mainly associated with host cell invasion and proinflammatory cell death [21,47]. Accordingly, during the systemic phase of infection and in *Salmonella*-infected macrophages in vitro, the SPI-1 gene expression is down-regulated [7,39,48]. Unlike SPI-1, the SPI-2 genes are induced in the systemic phase of infection, enabling bacteria to survive and replicate within macrophages [49,50]. Based on the intra-macrophage induced expression of RyhB-1 and RyhB-2 [7,8] and their negative effects observed on the expression of SPI-1-related genes (Figure 3), it is tempting to speculate that these sRNAs contribute to bacterial evasion of host cell recognition by the inflammasome through down-regulating the gene expression of some SPI-1 genes. Besides allowing the host cell invasion during the early phase of infection, SPI-1 is also involved in the induction of macrophage apoptosis [51]. In fact, mutant strains with continued expression of SPI-1 genes induces massive macrophage apoptosis by a mechanism involving caspases [52]. The rapid apoptosis of macrophage is likely to be involved in the escape of *Salmonella* from phagocytosis at early stages of infection. Nevertheless, once *Salmonella* has established a systemic infection, the excess macrophage apoptosis would be detrimental to the pathogen. Therefore, bacteria must suppress apoptosis sufficiently to allow time to replicate, escape, and invade new macrophages. Accordingly, the down-regulation of SPI-1 gene expression after epithelial cells invasion is essential for intracellular survival of *Salmonella* [21,53].

The expression analyses here reported, and several studies performed in *S*. Typhimurium [54], indicate that the roles of the *S.* Typhimurium RyhB sRNAs are largely overlapping. The common targets can be mainly explained by the highly conserved core region spanning nucleotides 37–69 [8]. However, differences in some regulatory targets have also been identified. For instance, RyhB-2 negatively regulates the expression of the STM1273 gene, which is encoded on the opposite strand [9]. Kim et al. (2013) [55] identified several motility genes (*fliF*, *flgJ*, and *cheY*), which are negatively regulated by RyhB-2, but not RyhB-1, while other gene targets (*acnB* and *safA*) are more strongly regulated by the RyhB-1. The ability to regulate the expression of distinct target mRNAs probably lies on the sequence divergence outside of the core region of these sRNAs. Although we did not observe differential targets in this study, we cannot rule out differences in other targets related to virulence. Despite the above, the evidence provided by our expression analyses from intracellular bacteria (Figure 3) suggests that *ryhB-1* seems to be epistatic over *ryhB-2* in some cases (e.g., *hilD*, *invF*, and *sipC* expression), while other results suggest that *ryhB-2* is epistatic over *ryhB-1* (e.g., *sopA* and *prgI* expression). Regarding these epistatic effects, in our previous study, characterizing the role of RyhB-1 and RyhB-2 in response to nitrosative stress, we observed that RyhB-1 was significantly up-regulated upon the pulse-expression of RyhB-2 [13]. Based on this information, we hypothesize that RyhB-2 have some regulatory effect on RyhB-1 expression and vice versa, which could explain, at least in part, the above-mentioned epistatic effects. However, the possibility of a regulation between the RyhB homologs needs to be further study. Concerning the expression analysis in the double mutant background, in most cases, no additive effect was observed on the expression levels of the targets as compared to the single mutants. We speculate that this could be the result of a compensatory response in the regulatory network to avoid an exacerbated deregulation of gene expression.

Although the usage of an *hfq*-deleted strain would be useful to fully confirm the direct interaction between the *trans*-encoded RyhBs and *sicA* and *rtsB* mRNAs in the two-plasmid assays, the heterologous expression of SPI-1 related genes of *S*. Typhimurium in *E. coli* strongly suggests a direct regulation by base-pairing. The noticeable down-regulation of the regulatory proteins SicA and RtsB by the RyhBs, evidenced in the expression analyses (Figure 3 and Figure 4), argues in favor of the overall regulatory effect observed on the other SPI-1 related genes. The invasion protein chaperone SicA acts together with InvF to activate the expression of SPI-1 and T3SS-related genes [56]. The regulation of RyhBs on SicA, and therefore, on the InvF-SicA complex, could lead to indirect gene expression regulation of *prgI*, *invI*, *sipA*, *sipB*, *sipC*, *sopA*, and *sopB*. On the other hand, the regulator RtsB is encoded in an operon along with RtsA, another regulatory protein. RtsA and RtsB coordinate induction of invasion and repression of motility [57]. RtsA induces expression of HilA, HilD, HilC, and InvF, all transcriptional factors of a complex regulatory network that activate SPI-1 genes and T3SS effectors required for the invasion stage of infection [21]. Thus, the down-regulation of RtsA by RyhBs could lead to indirect regulation of the transcriptional factors HilA, HilD, HilC, InvF, and their regulatory network. For its part, RtsB functions as a repressor of flagellar class 1 gene expression [57], by binding to the *flhDC* promoter region, and the significance of its down-regulation by RyhBs could lie in the subsequent activation of entire flagellar regulon allowing *Salmonella* escapes from apoptotic or oncotic macrophages [44,58]. Although RyhBs directly interact with the message of RtsB, the down-regulation of RtsA could resemble other cases of polycistronic mRNA regulation by sRNAs where a double-strand specific RNA endoribonuclease initiates a rapid degradation of the entire transcript [37]. Such is the case of the *sdhCDAB* mRNA in *E. coli*, which is down-regulated by interaction with RyhB at the translation start of the second cistron, *sdhD*. Even though the *sdhC* transcript is located upstream to the sRNA-mRNA pairing region, its levels are also decreased in the presence of the sRNA [37]. Although in silico analysis predicted favorable interactions between the sRNAs and the *rtsB* and *sicA* mRNAs, including relatively long base-pairing (Figure 4a), we cannot rule out the possibility of direct interaction with the other mRNAs here analyzed.

In summary, this study demonstrates that the sRNAs RyhB-1 and RyhB-2 contribute to the modulation of the *S.* Typhimurium virulence inside RAW264.7 macrophages by down-regulating the SPI-1 gene expression and participating in the intracellular growth restraint. Further studies with murine models will be useful to increase our understanding of RyhBs roles in *S*. Typhimurium virulence, determining their precise participation in acute and/or persistent infections.

## Data Availability

Not applicable.

## Supplementary Materials

The following are available online at https://www.mdpi.com/2076-2607/9/3/635/s1, Figure S1: Expression of the sRNAs RyhB-1 and RyhB-2 from intracellular bacteria infecting RAW264.7 macrophages, Figure S2: Microscopy of RAW264.7 cells infected with S. Typhimurium strains, Figure S3: Growth curves of S. Typhimurium ryhBs mutants in LPM, Figure S4: Expression of SPI-1- and metabolism-related genes from intracellular complemented strains infecting RAW264.7 macrophages, Table S1: Primers (Oligos) used in this study.
