# Supplementary material for: The Small RNA RyhB Homologs from Salmonella Typhimurium Restrain the Intracellular Growth and Modulate the SPI-1 Gene Expression within RAW264.7 Macrophages
Source: Microorganisms. 2021 Mar 18;9(3):635. doi: 10.3390/microorganisms9030635 (PMC8002944; doi:10.3390/microorganisms9030635)
Supplement: Supplementary file 1 [file microorganisms-09-00635-s001.pdf]

Supplementary Materials

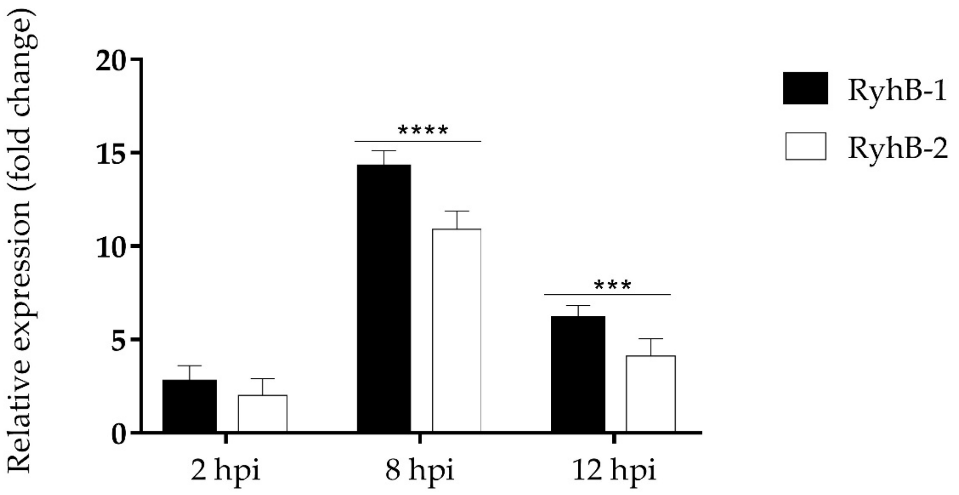

Figure S1. Expression of the sRNAs RyhB-1 and RyhB-2 from intracellular bacteria infecting RAW264.7 macrophages. RAW264.7 macrophages were infected with the wild type strain. Total RNA was extracted at 2, 8, and 12 hpi and the relative expression of the sRNAs was determined from intracellular bacteria by qRT-PCR. Values were normalized to the levels of the 16S rRNA. The relative expression of each sRNA of intracellular bacteria was calculated using the $2^{-\Delta\Delta CT}$ method and represented as the *n*-fold change relative to the wild type strain in the cell culture medium at stationary phase. Asterisks represent statistically significant differences with respect to the wild type in the cell culture medium (*** $p = 0.0001$; **** $p < 0.0001$). Data represent the means ± standard deviations ($n = 3$).

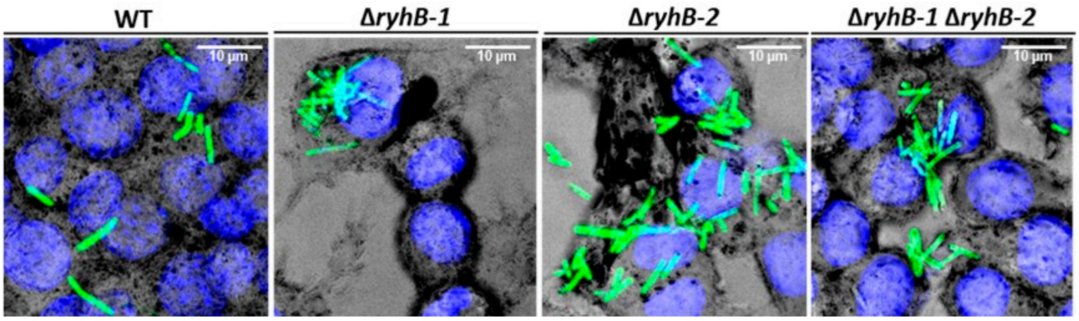

Figure S2. Microscopy of RAW264.7 cells infected with *S.* Typhimurium strains. RAW264.7 macrophages were infected with wild type (WT), Δ*ryhB-1*, Δ*ryhB-2*, and Δ*ryhB-1* Δ*ryhB-2* strains carrying the pDiGc plasmid for constitutive expression of GFP, and then visualized in a Leica TCS SP8 microscope by fluorescence microscopy at 8 hpi.

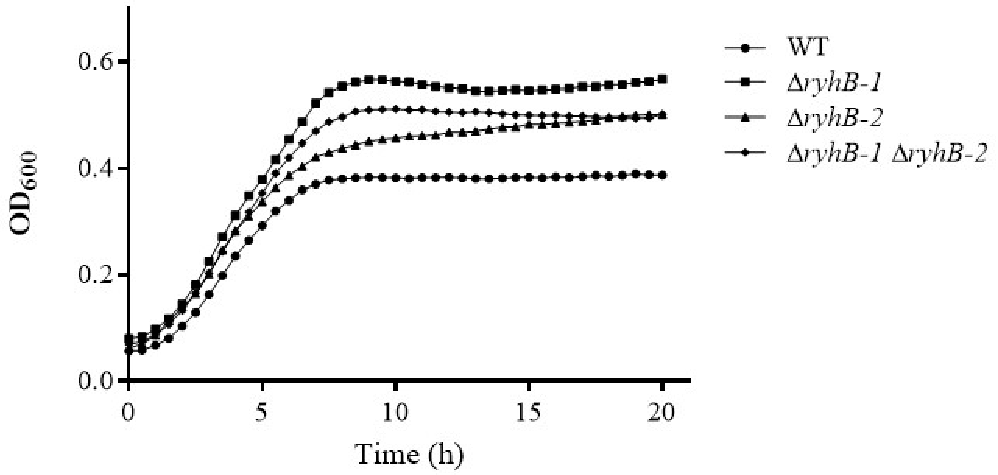

Figure S3. Growth curves of *S*. Typhimurium *ryhBs* mutants in LPM. Wild type (WT), Δ*ryhB-1*, Δ*ryhB-2*, and Δ*ryhB-1* Δ*ryhB-2* strains were grown in LPM and OD_{600} was measured at different time points. Data represent the means of three independent assays.

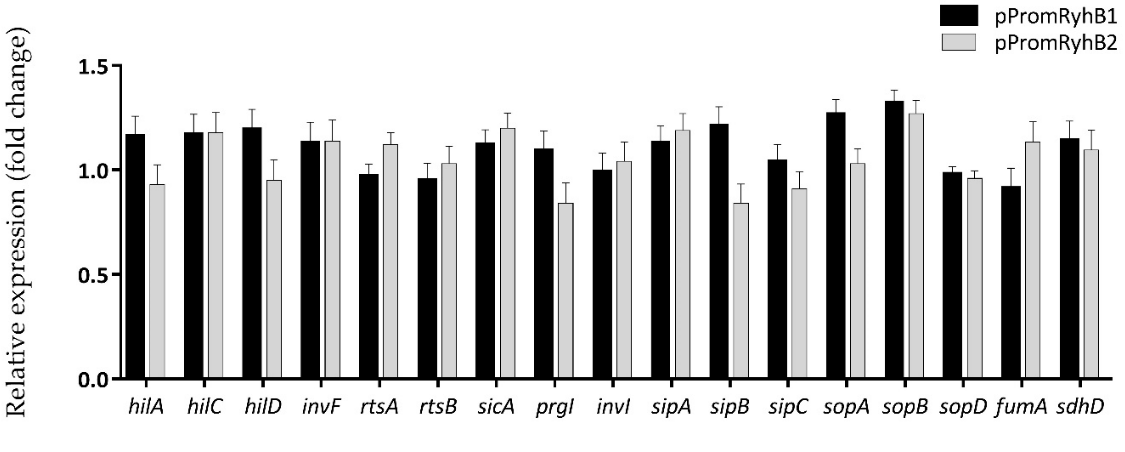

Figure S4. Expression of SPI-1- and metabolism-related genes from intracellular complemented strains infecting RAW264.7 macrophages. RAW264.7 macrophages were infected with wild type, Δ*ryhB-1* complemented (pPromRyhB1) and Δ*ryhB-2* complemented (pPromRyhB2) strains. Total RNA was extracted at 8 hpi and the relative expression of putative targets was determined from intracellular bacteria by qRT-PCR. Values were normalized to the levels of the 16S rRNA. The relative expression of each mRNA transcript of the complemented strains was calculated using the $2^{-\Delta\Delta CT}$ method and represented as the *n*-fold change relative to the wild type strain. Data represent the means ± standard deviations (*n*= 3).

Table S1. Primers (Oligos) used in this study

Name	Sequence 5´- 3´	Used for
pKD3/pKD4 Fw	TTTGCAAAAAGAAGTAGACAACTGCGAATGAGAATGATTGTAGGCTGGA GCTGCTTCG	Gene deletion
pKD3/pKD4 Rv	AGTTTGTTCACGGCAAGCGCGCAGGG CCCGGAGCGTACTACATATGAATATCCTCCTTAG	Gene deletion
ryhB-1_mut_Fw	GTTGCAATCATTAATGATAACGATTATCTTTATCAA TGTAGGCTGGAGCTGCTTCG	Gene deletion
ryhB-1_mut_Rv	CCATGAATTCGACAT GGGATAGATAGCGGTTAGCAACATATGAATATCCTCCTTAG	Gene deletion
ryhB-2_mut_Fw	ACCTATCGCCATGAACTATCGTGGCGA CGGAGGATGAATATGTAGGCTGGAGCTGCTTCG	Gene deletion
ryhB-2_mut_Rv	GT CGAAATGGCCATCCATTGCGCCACGGATGGCCTCTGC CCATATGAATATCCTCCTTAG	Gene deletion
ryhB-1_check_Fw	CTCGCTGAGAAAGAAAATT CC	Gene deletion
ryhB-1_check_Rv	CCTACAAAAGCAGATGCCTC	Gene deletion
ryhB-2_check_Fw	CATC GTCAGGAAAGTGAAGT	Gene deletion
ryhB-2_check_Rv	ACGTAAGGAGATTGTTC GTC	Gene deletion
ryhB-1_pBR322_Fw	CCGGGAATTCAACAACTATTGCT CGGGCGG	pBR322 cloning
ryhB-1_pBR322_Rv	GGCCGGATCCCCCAAAAAAA AAGCCAGCAAAAGC	pBR322 cloning
ryhB-2_pBR322_Fw	CGGGATCCTG CGTTTAGCTTTTGATTTTTC	pBR322 cloning
ryhB-2_pBR322_Rv	CGGAATTCA AAAAAAGCCCGCACTCGGT	pBR322 cloning
hilA Fw	CATTACTCTATCGTGAAGGGATT	qRT-PCR
hilA Rv	AGCGGGTTGGTGTTCTATCAA	qRT-PCR
hilC Fw	GGGAGTTCATCGAACGATCT	qRT-PCR
hilC Rv	CACTCATCCCTGGCAACAAG	qRT-PCR
hilD Fw	GTAAGTAATAGTCATCAGCGTCC	qRT-PCR
hilD Rv	TGAGCACCAACATCCCAGGT	qRT-PCR
invF Fw	GTTGTCGCACCAGTATCAGG	qRT-PCR
invF Rv	GTAACAGCGCCAGTACCTTAT	qRT-PCR
rtsA Fw	CAGGTGGGGAGCATTGAATG	qRT-PCR
rtsA Rv	CTTGCTACGCCTGTTTCTATTG	qRT-PCR
rtsB Fw	GAG ATA TCT GAC AAT GCA GTA TAA G	qRT-PCR
rtsB Rv	GAT ATG TAA AAC TCA TTC AGC ATC	qRT-PCR
sicA Fw	GTG TTG CGG AAA TGA TTT GGG	qRT-PCR
sicA Rv	CCA TGG TGT AAT CGG GAT TG	qRT-PCR

prgI Fw	CACCTTGGTCAGGCTATCTG	qRT-PCR
prgI Rv	GGAAGTTCTGAATAATGGCAGC	qRT-PCR
invI Fw	CCAGAATTAAAGTATTGCAGCG	qRT-PCR
invI Rv	CTGCCGGCGAACAATAGAC	qRT-PCR
sipA Fw	TAATGGAGTTTGCCGGGCTC	qRT-PCR
sipA Rv	CTCCTGCATAACAAGATTCGTC	qRT-PCR
sipB Fw	GCCAAGAGTGTTTATGACGCT	qRT-PCR
sipB Rv	GAATTTGGTCAGAATGTTATCCG	qRT-PCR
sipC Fw	TGCCGCGAATACGTTAATGCT	qRT-PCR
sipC Rv	CGCCTCTTTCATTCTGCAGC	qRT-PCR
sopA Fw	TCTAGGTGATATCTGGCTGCA	qRT-PCR
sopA Rv	CTTACCAGCGAACTTTCAACTG	qRT-PCR
sopB Fw	CAGCTTAATAACCAGCCCTG	qRT-PCR
sopB Rv	TTTACCGTCCTCATGCACAC	qRT-PCR
sopD Fw	ATGCGCTGGAAGTGTTACACT	qRT-PCR
sopD Rv	ATTGCTTTGGCTGATCACCGT	qRT-PCR
fumA Fw	AGGTGGCGGATATTCTGCGC	qRT-PCR
fumA Rv	CACCAGTCCAGACGCGTTGAC	qRT-PCR
sdhD Fw	GTGCTACCGCTATCGTTCTGA	qRT-PCR
sdhD Rv	CTTCAAAGGTCAGTTCGCCG	qRT-PCR
q16SFw	TACCTGGTCTTGACATCCAC	qRT-PCR
q16SRv	TTATCACTGGCAGTCTCCTT	qRT-PCR
pSF-p15A-rpsL_ rtsB Fw	ATCTAAGCTTTTTGTTGTATGACTGGGG	*rpsL* promoter cloning for *rtsB* constitutive expression
pSF-p15A-rpsL_ rtsB Rv	TATCGAATTCCACCCAGCCCTCTCACC	*rpsL* promoter cloning for *rtsB* constitutive expression
pRtsB Fw	ATCTGAATTCGTTAATTGAGATATCTGACA	*rtsB* cloning for constitutive expression
pRtsB Rv	TATCTCTAGATTACGTAATATCGACTGAT	*rtsB* cloning for constitutive expression
pSF-p15A-rpsL_ sicA Fw	ATCTAAGCTTTTTGTTGTATGACTGGGG	*rpsL* promoter cloning for *sicA* constitutive expression

pSF-p15A-rpsL_ sicA Rv	TATCGAATTCGCGCATTCTAGTTAGGCT	*rpsL* promoter cloning for *sicA* constitutive expression
pSicA Fw	ATCTGAATTCATCAGATAAACGCAGTCGT	*sicA* cloning for constitutive expression
pSicA Rv	TATCTCTAGATTATTCCTTTTCTTGTTCA	*sicA* cloning for constitutive expression
pBAD_ryhB-1 Fw	5′P-GCATTCAGGGGAACCCCTAC	pBAD cloning
pBAD_ryhB-1 Rv	GTTTTTTTCTAGAAAAAAAAAGCCAGCAAAAGCT	pBAD cloning
pBAD_ryhB-2 Fw	5′P-TACCGAGTGGTTGAGTTTAT	pBAD cloning
pBAD_ryhB-2 Rv	GTTTTTTTCTAGAAAAAAAAAGCCCGCACTCGGT	pBAD cloning
pBADcontrolFw	GGAGAAACAGTAGAGAGTTGC	pBAD cloning
pBADcontrolRv	TTTTTTCTAGATTAAATCAGAACGCAGA	pBAD cloning
pRyhB1^{MUT1}_Fw	CTGAAAGCACGACATTGCTGCATTGCTTCCAGTAT	site-directed mutagenesis
pRyhB1^{MUT1}_Rv	ATACTGGAAGCAATGCAGCAATGTCGTGCTTTCAG	site-directed mutagenesis
pRyhB1^{MUT2}_Fw	CTGAAAGCACGACATTGCTCACATTGCTTCCAGT	site-directed mutagenesis
pRyhB1^{MUT2}_Rv	ACTGGAAGCAATGTGAGCAATGTCGTGCTTTCAG	site-directed mutagenesis
pRyhB2^{MUT1}_Fw	TGTTCGGTACGACATTGCTGCATTGCTTCCAGTAT	site-directed mutagenesis
pRyhB2^{MUT1}_Rv	ATACTGGAAGCAATGCAGCAATGTCGTACCGAACA	site-directed mutagenesis
pRyhB2^{MUT2}_Fw	TGTTCGGTACGACATTGCTCACATTGCTTCCAGT	site-directed mutagenesis
pRyhB2^{MUT2}_Rv	ACTGGAAGCAATGTGAGCAATGTCGTACCGAACA	site-directed mutagenesis
pRtsB^{MUT}_Fw	TTAATTGAGATATCAACACATGCAGTATAAGAAC	site-directed mutagenesis
pRtsB^{MUT}_Rv	GTTCTTATACTGCATGTGTTGATATCTCAATTAA	site-directed mutagenesis
pSicA^{MUT}_Fw	CAGATAACAGGAGTACAACATGGATTATCAAAAT	site-directed mutagenesis
pSicA^{MUT}_Rv	ATTTTGATAATCCATGTTGTACTCCTGTTATCTG	site-directed mutagenesis
